# Biotechnological Potential of *Bdellovibrio* and Like Organisms and Their Secreted Enzymes

**DOI:** 10.3389/fmicb.2020.00662

**Published:** 2020-04-15

**Authors:** Eleni Bratanis, Tilde Andersson, Rolf Lood, Ewa Bukowska-Faniband

**Affiliations:** Division of Infection Medicine, Department of Clinical Sciences, Lund University, Lund, Sweden

**Keywords:** predatory bacteria, biotechnology, biocontrol, antibody modification, antibiotic resistance, biofilm

## Abstract

*Bdellovibrio* and like organisms (BALOs) are obligate predatory bacteria that selectively prey on a broad range of Gram-negative bacteria, including multidrug-resistant human pathogens. Due to their unique lifestyle, they have been long recognized as a potential therapeutic and biocontrol agent. Research on BALOs has rapidly grown over the recent decade, resulting in many publications concerning molecular details of bacterial predation as well as applications thereof in medicine and biotechnology. This review summarizes the current knowledge on biotechnological potential of obligate predatory bacteria and their secreted enzymes.

## *Bdellovibrio* and Like Organisms (BALOs)

Predation is a natural and essential interaction present at all trophic levels and in all ecosystems, contributing to maintenance of ecological balance (Johnke et al., 2019). While predation as a term often is associated with larger animals hunting and feeding upon prey, the same definition is true even for microorganisms. Predatory bacteria can be found within a broad taxonomy, including both facultative and obligate predators, defined by their feeding behavior. Whilst obligate predators survive by consuming prey cells, facultative predators readily switch to a saprophytic lifestyle, consuming a wide array of substrates in the absence of appropriate prey (Jurkevitch, 2007; Korp et al., 2016). To date, an obligatory predatory lifestyle is limited to α-proteobacteria (genus *Micavibrio*) and δ-proteobacteria (families: *Bdellovibrionaceae, Bacteriovoraceae, Peredibacteraceae*, *Halobacteriovoraceae*, and *Pseudobacteriovoracaceae*), all classified under the umbrella terminology *Bdellovibrio* and like organisms (BALOs) (Rotem et al., 2014; Koval et al., 2015; McCauley et al., 2015; Paix et al., 2019). Even though obligate predatory bacteria were first described nearly 60 years ago, many of the molecular mechanisms of prey invasion, nutrient acquisition, as well as details on the extent and importance of bacterial predation, remain limited and rather elusive. Until recently, the progress within this field of research has long remained rather insignificant. However, predatory bacteria are now gaining increased attention, much owed to the alarming reports on the rise in antimicrobial resistance (AMR) and a general rise in environmental awareness. Several reports have proposed and demonstrated the potential use of predatory bacteria as live antibiotics, water clean-up and biocontrol agents, as well as being sources for the discovery of novel biotechnological tools for research (Yair et al., 2009; Pérez et al., 2016). *Bdellovibrio bacteriovorus* is among the best-studied BALOs, and serves as a model organism for bacterial predation. *B. bacteriovorus* was first identified in the 1960s and was quite accurately described as a small parasite, and obligate predator of Gram-negative bacteria (Stolp and Starr, 1963). Continued characterization of *B. bacteriovorus* has since then confirmed these first reports. It was further described as a highly motile, δ-proteobacterium that employs an endobiotic (periplasmic) hunting strategy which entails the invasion of, and proliferation within, the periplasm of Gram-negative bacteria. Importantly its prey range includes several known human pathogens that either already have acquired, or are at great risk of acquiring resistance to antibiotics, such as enterohemorrhagic *Escherichia coli*, *Helicobacter pylori*, *Klebsiella pneumoniae*, *Pseudomonas*, and *Salmonella* (Sockett, 2009; Dashiff et al., 2011; Woodford et al., 2011; Dwidar et al., 2012b; Shatzkes et al., 2016). Other BALOs, including *Bdellovibrio exovorus* and *Micavibrio aeruginosavorus* employ an epibiotic strategy of predation, in which the predator remains attached to the prey cell and consumes it from the outside before dividing into two daughter cells (Jurkevitch, 2007; Pasternak et al., 2014; Pérez et al., 2016). Recent genomic analysis comparing periplasmic and endobiotic predators revealed that protein coding genome of epibiotic predators contained far fewer genes coding for lytic enzymes, limiting the interest in these predators for therapeutic and/or biotechnological applications (Pasternak et al., 2014). There are additional characteristics that limit the potential applications of *B. exovorus* and *M. aeruginosavorus*. These include a restricted prey range as compared to *B. bacteriovorus*, the inability to grow in the absence of prey, limiting product shelf life, and displayed resistance toward multiple antibiotics including ampicillin, kanamycin, chloramphenicol, carbapenems, and polymyxins (Koval et al., 2013; Pasternak et al., 2014).

**FIGURE 1:**
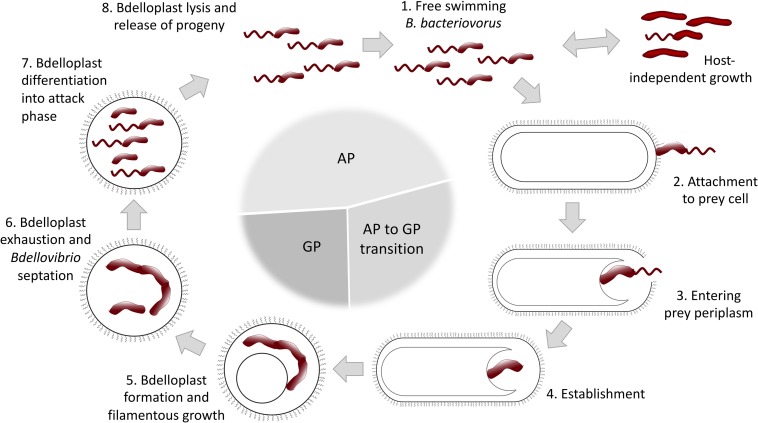
Schematic representation of *B. bacteriovorus* life cycle (for a detailed description see text). AP, attack phase; GP, growth phase.

*B. bacteriovorus* lifecycle has long been described to be biphasic, divided into a free-living attack phase (AP) and an intraperiplasmic growth phase (GP) (Figure 1). However, a third AP to GP transition phase, where prey-derived cues trigger a specific bdellovibrio transcription profile, was recently introduced (Rotem et al., 2015). In the AP *B. bacteriovorus* collides with and attaches to Gram-negative prey cells. It invades into host by creating a pore in the outer membrane and crossing the peptidoglycan layer, to finally establish itself within the prey periplasm. Collision with the prey occurs seemingly at random, and it has been suggested that the predatory cell remains reversibly attached for a brief “recognition” period before becoming irreversibly anchored (Burnham et al., 1968; Rendulic et al., 2004; Lambert et al., 2016). Successful recognition triggers the aforementioned transition to an intermediate phase that facilitates invasion into the host cell and formation of an osmotically stable niche, protected from phage attacks, photooxidation and pollutants, called bdelloplast (Friedberg, 1977; Markelova, 2002; Yair et al., 2009). It has been proposed that *B. bacteriovorus* uses its type IV pili to pass through the membrane, then sheds the flagellum and reseals the pore after entering the prey. Sensing of a second prey cue facilitates transition to the GP and filamentous growing. Bdelloplast formation causes a distinct rounding up of the usually rod-shaped prey cell, resulting from peptidoglycan cell wall modifications. This modification has been shown to prevent self-competition between individual predators for the same prey and promote 1:1 predator to prey ratio. When the prey is exhausted, the predator divides by septating into several flagellated progeny cells, followed by host cell lysis and progeny release, whereupon the cycle begins anew (Tudor et al., 1990; Rendulic et al., 2004; Evans et al., 2007; Karunker et al., 2013; Lambert et al., 2015; Avidan et al., 2017).

Despite being primarily recognized as an obligate predatory bacterium, *B. bacteriovorus* can switch into a host-independent (HI) lifestyle (Figure 1), displaying either saprophytic (requiring prey extracts) or axenic (growing on complete media without prey components) growth. The initial events involved in lifestyle switching from host-dependent (HD) to HI growth are associated with mutations in the growth control circuitry. HI mutants are readily isolated from environmental samples (Diedrich et al., 1970; Doskina, 1973; Hobley et al., 2012b), but the extent of this phenomenon is yet to be determined. HI strains display dimorphic growth, and they maintain their predatory capabilities if regularly grown in the presence of prey. Studies investigating changes in gene expression between HI- and obligate predatory *B. bacteriovorus* have revealed distinct up- and down regulations of specific genes in the two life-styles (Reiner and Shilo, 1969; Cotter and Thomashow, 1992a; Dori-Bachash et al., 2008; Lambert et al., 2010; Chang et al., 2011; Roschanski et al., 2011).

BALOs are ubiquitous in a wide variety of manmade and natural environments. These include soil and different aquatic habitats such as rivers, lakes, the open ocean, sewage and wastewater treatment plants (WWTPs) (Richardson, 1990; Jurkevitch et al., 2000; Sockett, 2009; Hobley et al., 2012b; Oyedara et al., 2016; Paix et al., 2019). Bdellovibrios have also been recovered from the gills of blue crabs and oysters, and more recently from mammalian feces and the mammalian gastrointestinal tract (Kelley and Williams, 1992; Schwudke et al., 2001; Rotem et al., 2014). Naturally, the potential use of live bacteria as therapeutics raises concerns regarding the safety and efficacy of BALOs administration. This aspect has been, and continues to be, thoroughly investigated using both human cells and numerous animal models such as zebrafish, mice, rats, rabbits, guinea pigs, and chicks. The results demonstrate an inability of *B. bacteriovorus* and *M. aeruginosavorus* to invade mammalian cells, and no apparent pathological effects or signs of cytotoxicity or reduction in cell viability, supporting the proposition that these two BALOs are inherently non-pathogenic to mammals (Westergaard and Kramer, 1977; Atterbury et al., 2011; Dwidar et al., 2012b; Gupta et al., 2016; Willis et al., 2016; Shatzkes et al., 2015, 2016, 2017b). It has even been suggested that *B. bacteriovorus* may contribute to health as part of the human gut microbiota (Iebba et al., 2013). Although considered as obligately aerobic bacteria, research has shown that BALOs can survive under anoxic conditions and that certain strains, including *B. bacteriovorus*, are able to grow and attack under microaerobic conditions (Kadouri and Tran, 2013; Patini et al., 2019). These findings further support the potential of using these predators as therapeutics in environments such as the gastrointestinal tracts (Sockett and Lambert, 2004; Dwidar et al., 2012b). Studies using an *in vivo* airway infection model demonstrated that both *B. bacteriovorus* and *M. aeruginosavorus* could reduce the burden of *K. pneumoniae* in rat lungs without any adverse effects on lung pathology, indicating that the potential therapeutic application of BALOs is not limited to the gut. This investigation was further expanded to evaluate the efficacy of intravenous administration. The results clearly showed an inability of *B. bacteriovorus* and *M. aeruginosavorus* to reduce *K. pneumoniae* burden in blood or prevent dissemination to other organs, suggesting that predatory bacteria might not be an effective treatment option for blood infections (Shatzkes et al., 2015, 2016). A later study by Russo et al. (2018) demonstrated that *B. bacteriovorus* treatment is also effective in *Yersinia pestis* infection of mouse lungs.

One concern with regards to the applicability of bdellovibrios as therapeutics is the development of prey resistance and incomplete eradication of prey. This phenomenon is well known and has been widely reported in the literature. Plastic prey resistance has been described as a phenotypic response to stress, rather than a mutational event. The phenomenon is thought to be a common event in the environment to ensure the survival of both the prey and the predator. However, the prey susceptibility to predation seems to be regained upon continued culturing. Thus, it has been argued that despite the display of some prey resistance, the number of resistant prey will be low, making bdellovibrios an effective therapeutic or biocontrol agent (Shemesh and Jurkevitch, 2004; Sockett and Lambert, 2004; Jurkevitch, 2007). It has also been suggested that permanent mutation-based resistance can emerge, however, it is still ill-defined (Varon, 1979; Gallet et al., 2007, 2009). The natural resistance of bdellovibrios to β-lactam antibiotics also opens up the possibility for treatments using these bacteria in conjunction with penicillin (Sockett and Lambert, 2004; Dwidar et al., 2012b).

The revived and growing interest in BALOs and other predatory bacteria has resulted in a rapid increase of information and knowledge that is now readily available. This includes a better overview and understanding of the large diversity of predatory species and their widespread distribution, as well as genome, proteome, secretome, and biochemical data (Schwudke et al., 2001; Rendulic et al., 2004; Dori-Bachash et al., 2008; Song et al., 2009; Pan et al., 2011; Karunker et al., 2013; Avidan et al., 2017; Bratanis et al., 2017; Bratanis and Lood, 2019). Although much of the interest in BALOs relates to the use of predatory bacteria as biological-based therapeutic agents (Willis et al., 2016; Negus et al., 2017), their application is more versatile, as shown by the already existing products on the market. The Chinese Hebei Weierli Animal Pharmaceutical Group produces *B. bacteriovorus* as a probiotic for aquatic animals, poultry, and farm animals (Qi et al., 2009), and the Canadian company GeneBio Systems Inc. produces a range of recombinant *B. bacteriovorus* proteins. In this review, we will focus the discussion on the potential of *B. bacteriovorus* and its secreted enzymes as biotechnological tools within different fields.

## *B. bacteriovorus* Secretome as a Source of Novel Biotechnological Tools

When considering the predatory lifestyle of *B. bacteriovorus*, the requirement of a large variety of enzymes and transporters becomes evident. This necessity is further emphasized by the fact that *B. bacteriovorus* might not be able to synthesize some of the amino acids required for protein synthesis, thus making the predator highly dependent on the uptake of degraded host products (Rendulic et al., 2004; Barabote et al., 2007). The availability of the complete genome sequence (approximately 3.8 Mb) of *B. bacteriovorus* HD100 has provided the basis for further analysis of its genome, predicted to contain >3580 open reading frames (ORFs), with many of those being part of the secretome (Rendulic et al., 2004). A comparison of 176 theoretical bacterial secretomes (i.e., all proteins with predicted N-terminal signal sequence) showed that Gram-negative bacteria on average contain a larger number of potential Sec-dependent sequences, and *B. bacteriovorus* HD100 had the largest secretome: 42.4% (1520 proteins) amongst the bacteria included in the study (according to the authors’ criteria) (Song et al., 2009). The vast majority of these putatively secreted proteins have unassigned functions; likewise many are membrane-associated or lipoproteins. If all those are disregarded, the number of free secreted proteins becomes 222. Approximately half of them are predicted to be hydrolytic enzymes. The original bioinformatics study by Rendulic et al. (2004) showed that the *B. bacteriovorus* HD100 genome encodes an estimated 293 potential lytic proteins including 150 annotated proteases and peptidases, 10 glycanases, 20 DNases, 9 RNases, and 15 lipases. However, only 15 of these were mentioned as potentially extracellular. An additional study performed in a HI *B. bacteriovorus* HI-6 strain identified 59 proteins in the secreted proteome, out of which 50 contained a signal peptide (Dori-Bachash et al., 2008). Fourteen of these proteins resembled known enzymes including several serine proteases, some of which were further analyzed and identified as two trypsin-like enzymes, one V8-like Glu-specific endopeptidase and one carboxypeptidase. The putative V8-like endopeptidase has since been identified and characterized in more detail as a serine protease, BspK (Bratanis et al., 2017).

The genome of *B. bacteriovorus* HD100 has been predicted to encode a large number of different systems for both cytoplasmic and outer membrane protein transportation (Rendulic et al., 2004). The comprehensive bioinformatic analysis by Barabote et al. (2007), revealed the presence of at least four types of inner membrane secretion systems and five types for outer membrane secretion. Interestingly, deletion studies targeting the twin-arginine transport system (Tat) in *B. bacteriovorus* have shown to be detrimental for both HD and HI growth (Chang et al., 2011). In addition to the Tat system, *B. bacteriovorus* also relies on the type I and II (Sec) secretion systems, whilst the type III, IV and VI secretion systems, associated with bacterial virulence, are absent (Rendulic et al., 2004; Barabote et al., 2007; Rotem et al., 2014).

While the functions of many proteins are yet to be elucidated, the overall *B. bacteriovorus* secretome has shown to be extremely dynamic, revealing cell cycle-dependent functions of many proteins. Thus, in regards to its arsenal of hydrolytic enzymes, *B. bacteriovorus* should be considered an interesting biological source for identifying novel bacterial proteins with applications within basic research and the life science industry.

### BALOs Proteases as Novel Antibody-Modulating Tools

The rapid development of biopharmaceuticals, and antibody-based therapeutics in particular, has generated a need for novel biotechnological tools and innovative methods to ensure product quality and safety. Antibodies are natural, exceptionally heterogeneous molecules, both in regards to the protein backbone and potential post-translational modifications including *N*- and *O*-glycosylation, deamination and chain trimming. This inherent antibody heterogeneity and sensitivity in monoclonal antibodies (mAb) production emphasize the need for reproducible and reliable methods for analysis and quality control. These analyses are commonly performed by mass spectrometry (MS) -based methods. The sensitivity of MS analysis is increased by fragmentation of intact proteins, preferentially into overlapping peptides, highlighting the need for a larger selection of biotechnological tools for this purpose (Aebersold and Mann, 2003; Gupta et al., 2010; Hansel et al., 2010). This creates a need for hydrolytic enzymes displaying unique and complementary cleavage profiles, in addition to currently marketed enzymes used for antibody analysis. This makes BALOs, with their plethora of hydrolytic enzymes, an interesting potential source for the identification of such tools.

The application of bacterial enzymes as biotechnological tools for antibody analysis is since long common practice, and two examples of commercially available biotechnological tools used for specific hydrolysis of human IgG are the streptococcal enzymes IdeS and EndoS (Genovis AB). Interestingly, *B. bacteriovorus* is now emerging as a new source for the identification of novel enzymes with biotechnological potential. This potential can be exemplified by the identification and characterization of BspK and BspE with described enzymatic activities on human antibodies (Bratanis et al., 2017; Bratanis and Lood, 2019). BspK specifically hydrolyzes IgG_1_ (most common therapeutic antibody) in the hinge, enabling middle-down MS analysis of the biological therapeutic (Bratanis et al., 2017). Similar enzymes (e.g., Ides, SpeB) are currently being used within the biopharma industry for such purposes. BspE specifically hydrolyzes the Fc-tail from IgA, with its glycan attached (Bratanis and Lood, 2019). While IgA is not commonly used for the development of therapeutic antibodies, BspE is still a valuable tool for the basic research of IgA, Fc-interactions and complement activation – findings that may eventually be translated into products. The rapid development and increasing number of approved mAb on the market creates a need and incentive to identify and characterize novel antibody degrading or modifying proteins.

## Predatory Bacteria as a Biocontrol Agent

In both natural and man-made habitats, contamination of microorganisms can sometimes have detrimental outcomes. For instance, *Vibrio cholerae* in lake water is a major cause of morbidity and mortality in parts of Africa (Bwire et al., 2017), and bacteriophage infections appear to negatively influence the performance of WWTPs (Fu et al., 2009; Barr et al., 2010). Where conventional methods for removal of contaminating and/or pathogenic microorganisms fail, biocontrol agents might constitute a viable alternative. Biological control, meaning the use of any organism to target an undesirable population of another, is a technique being increasingly recognized for its low cost and limited adverse effects on the environment, wildlife and public health (Kergunteuil et al., 2016).

It follows that the idea of using predatory bacteria as a biocontrol agent is also gaining momentum. For example in poultry farming where the birds are recognized as a primary source of *Salmonella*, yearly affecting millions of people worldwide. Strategies to prevent salmonellosis include good agricultural practices combined with additional prevention measures (OIE - World Organisation for Animal Health, 2019), to which predatory bacteria could potentially be added. It has been shown that orally administered *B. bacteriovorus* is able to effectively manage *Salmonella* infections in young chicks, without adverse effects on the chick’s health and well-being (Atterbury et al., 2011). It has also been proposed that *B. bacteriovorus* might be useful in the freshwater farming industry as a biological control agent of the shrimp pathogens *V. cholerae* (Cao et al., 2015) and *V. parahaemolyticus* (Kongrueng et al., 2017). The same group subsequently showed that *Bdellovibrio* could be prepared as an encapsulated powder and stored at room temperature over several months for later use as a biodisinfectant in shrimp aquaculture (Cao et al., 2019).

BALOs have several prospective applications also in agriculture. Introduced or naturally occurring strains, cultured at large scale, could for instance be used as a wide-spectrum biocontrol agent combating phytopathogens that would otherwise damage the crops (Scherff, 1973; Jurkevitch et al., 2000; McNeely et al., 2017; Youdkes et al., 2020). The ensuing process of food spoilage might also be mitigated through predation. Saxon et al. (2014) showed that *B. bacteriovorus* is able to eliminate *Pseudomonas tolaasii*, a problematic pathogen of cultured mushrooms. Administration of *B. bacteriovorus* on the surface of post-harvest mushrooms resulted in the reduction of brown-blotch lesions, which could help extend the shelf-life of the product (Saxon et al., 2014). Similarly, Ottaviani et al. (2019) demonstrated that *B. bacteriovorus* is able to control *E. coli* and other spoilage bacteria in meat products. This preliminary study showed that predatory bacteria can complement current methods of food spoilage prevention, as well as be a natural alternative to preservatives and antioxidants. It has also been suggested that predatory species can be used later on in the food manufacturing process as a way of removing bacteria from processing equipment (Fratamico and Cooke, 1996).

In light of the current energy crisis, research centered around microalgae-derived biofuel is picking up and, by many, considered a promising alternative. However, the growth of microalgae in open ponds is often affected by bacterial contamination. Li et al. (2018) demonstrated that a *Bdellovibrio* sp. limited the number of contaminating bacteria, thereby promoting microalgae growth and the production of green biofuel. Another pressing environmental and economical concern is the amount of “waste activated sludge” generated by WWTPs. Waste activated sludge is the excess microorganisms that need to be removed to maintain balance within the biological system. Several studies have indicated that bacterial predation, in combination with environmental factors such as regulation of dissolved oxygen concentrations, is a key factor in limiting the production of waste activated sludge (Niu et al., 2016; Semblante et al., 2017). The volume of activated sludge can also be reduced by, e.g., release of intracellular water, accounting for 70–80% of the packed cell mass. It has been demonstrated that the treatment of activated sludge with *B. bacteriovorus* effectively improved its dewaterability in a dose dependent manner (Yu et al., 2017).

BALOs could furthermore reduce the turnover of pipeline steel in major cities and companies around the world by inhibiting the microbiologically influenced corrosion caused by sulfate-reducing bacteria (Qiu et al., 2016). As several of their potential biocontrol applications could help ease human footprint, BALOs research is of high value and very much in line with current political trends.

## Biofilm Formation and Degradation by BALOs

Environmental bacteria often exist as structured single- or multi-species communities attached to surfaces, with a cover of extracellular polymeric substances – also called biofilms. These biofilms can be found everywhere we find bacteria in the environment, but also on industrial equipment, WWTPs, and medical instruments (e.g., implants, shunts, and hospital surfaces). This is thus a problem stretching over several different fields of research, with different challenges. Here, we will discuss the role of BALOs in the formation and clearance of biofilms.

### BALOs Self-Formation of Biofilms

Despite its unique life cycle, the predatory bacterium *B. bacteriovorus*, like most bacteria, shares the ability to form biofilms. However, opposite to most bacteria, *B. bacteriovorus* has only been reported to form biofilms as HI mutants in nutrient-rich environments (Medina and Kadouri, 2009). As such, the addition of prey or lowering of nutrient accessibility results in phenotypic changes and detachment of the biofilm (Medina and Kadouri, 2009). A similar phenomenon was described by Ferguson et al. (2014), with *B. bacteriovorus* forming spatially organized communities of differentiated bacteria, with a central core of predatory active bacteria, and an outer morphologically heterogeneous community of HI cells. The presence of nutrient and lack of access to prey favored the diversification, and it was speculated that this phenotypic change resembling a biofilm may benefit BALOs persistence in the environment. Similarly, Williams et al. (2009) demonstrated that BALOs (*Bacteriovorax*) can form biofilms on oyster shells *in vitro*. Such formation was highly regulated by environmental factors (e.g., salinity, temperature, time) and allowed for longer survivability of BALOs as compared to planktonic cells, and biofilm formation was thus speculated to support survivability of BALOs in its aquatic habitats. However, although biofilm formation of BALOs has been reproduced *in vitro*, the natural occurrence and putative biological role remains to be investigated. Based on their findings, Williams et al. (2009) hypothesize that biofilms formed by HI cells are likely of high importance for the long-term presence of naturally occurring BALOs in several environments of biotechnological importance (e.g., WWTPs), serving as reservoir of predators. Finally, other putative applications may constitute preventive coating of surfaces with designated HI mutants to reduce ability of other bacteria to form biofilms.

### BALOs Regulation of Prey and Non-prey Biofilms

Rather than its ability to form self-biofilm, it is the ability of BALOs to inhibit formation, as well as reduce preformed biofilms of other bacteria, that has raised general interest. Several methods have been developed to specifically study the role of predatory bacteria in biofilms; including fluorescently labeled *B. bacteriovorus* (Mukherjee et al., 2015), chip calorimetry assays measuring metabolic heat during biofilm removal (Buchholz et al., 2012), as well as atomic force microscopy for more mechanistic insight in biofilm formation and degradation (Núñez et al., 2005); the latter being limited to single-layered structures.

Though being an exclusive predator of Gram-negative bacteria, even Gram-positive biofilms are prone to degradation by BALOs. The presence of Gram-positive biofilms induces an intracellular transcriptome response in *B. bacteriovorus*, different from that when exposed to planktonic cells, leading to secretion of several proteases (e.g., Bd2269 and Bd2692) (Im et al., 2018). Through the plethora of secreted enzymes, in particular its proteases and nucleases, *B. bacteriovorus* has the ability to both inhibit the formation of, as well as reduce preformed, biofilms of Gram-positive bacteria (Monnappa et al., 2014). The hydrolase secretion generates free monomers of macromolecules (e.g., amino acids and carbohydrates) from the Gram-positive biofilms, leading to a significant increase in ATP availability for BALOs (Im et al., 2018). Therefore, despite being unable to infect and prey upon Gram-positive cells, BALOs may still be able to benefit directly, and affect the Gram-positive microbiota due to their secretome.

Of special interest is the ability of BALOs to disrupt biofilms of medically relevant pathogens (Sun et al., 2017), as well as possibility to use them synergistically with certain antibiotics (e.g., ciprofloxacin) (Chanyi et al., 2016). However, the removal of environmental and industrial biofilms with BALOs has also been carefully investigated. While being highly efficient even at low multiplicity of infection and short incubation times, prolonged incubations (24 h) are more efficient resulting in >4 log reduction of viable bacteria within biofilms (Kadouri and O’Toole, 2005). Interestingly, further incubation, or addition of more BALOs do not result in additional clearing of bacteria in the biofilm. Possibly such failure to lyse further cells (as seen in planktonic cultures) is due to the presence of dormant biofilm cells (e.g., non-metabolically active persister cells), highly encapsulated cells, and/or plasticly resistant cells (Shemesh and Jurkevitch, 2004). Thus, while complete eradication of biofilms using BALOs may be doubtful, it may still allow for a significant reduction of biofilm bacteria in environmental, medical, and industrial settings.

For improved degradation of biofilms, it has also been suggested to combine CO_2_ treatment with BALOs on silicon chips (Dwidar et al., 2012a). Not only did such an approach result in an increased biofilm removal, but also limited the exposure of pathogen containing aerosols with live bacteria. Further, the addition of enzymes in combination with BALOs may also significantly affect biofilm degradation. The addition of specific carbohydrate hydrolases (e.g., poly-*N*-acetylglucosaminidases) has been shown to increase the ability of BALOs to degrade biofilms (Dashiff and Kadouri, 2011). However, the presence of proteinase K significantly reduces the ability of BALOs to prey upon biofilms (Dashiff and Kadouri, 2011). Likewise, despite DNA being a critical component of the biofilm, isogenic BALOs DNase mutants have shown a reduced ability to form self-biofilms, while being more efficient in clearing prey biofilms (Lambert and Sockett, 2013). Several suggestions have been raised to explain this incongruity, including DNA facilitating entrapment of BALOs within the biofilm, thus leading to increased predation of the cells.

In WWTPs bacteria often exist as communities within flocs of activated sludge, where they form multispecies biofilms. Floccular activated sludge has a function in the degradation of organic matter as well as the removal of nitrogen and phosphorus. Feng et al. (2016, 2017) demonstrated that these biofilms do not protect sensitive species from BALOs predation, with BALOs being able to penetrate and/or degrade the biofilm for access to its prey. Thus, the vast majority of Gram-negative bacteria within the sludge are sensitive to BALOs, irrespective of if they are planktonic or in biofilm. This bacterial predation may have a detrimental impact on the performance of WWTPs, which is discussed further in the section “Potential limitations.” However, with optimization, BALOs predation may instead help alleviate the issue of membrane biofouling during water treatment process, which is largely caused by an unsought for buildup of biofilm (Kim et al., 2013).

## Predatory Bacteria as a Strategy to Combat Horizontal Gene Transfer

**FIGURE 2:**
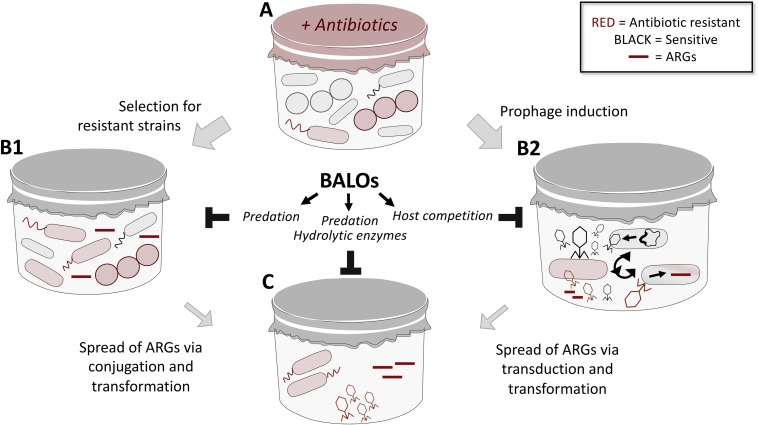
An overview on the mechanisms of spread of ARGs in mixed microbial communities (e.g., in WWTPs) and suggested role of predatory bacteria in limiting thereof. **(A)** Microbial communities that consist of both susceptible and resistant bacteria are exposed to a variety of stressors (e.g., antibiotics) in the environment, leading to selective pressure and expansion of resistant populations **(B1)**. BALOs may predate on such resistant population to reduce the ARGs pool. Antibiotics can also trigger induction of prophages that are capable of transduction as well as the release of resistance genes **(B2)**. Through host competition, i.e., BALOs preying on bacterial cells also targeted by phages for propagation, the number of phages carrying resistance genes will be limited. The expanding population of resistant bacteria **(B1)**, as well as the mobilized resistome **(B2)** are capable of spreading resistance via conjugation, transformation and transduction events **(C)**. Besides the ability of BALOs to limit spread of resistance through predation of bacterial cells (limit conjugation), its plethora of hydrolytic enzymes can lead to nucleolytic degradation of cell-free DNA (limit transformation) and inactivation of phage particles through action of secreted proteases (limit transduction); thus dictating the outcome of spread.

Antimicrobial resistance is posing a major threat to public health and is regarded as an important global problem to confront. While novel experimental means to kill resistant bacteria have been developed, including bacteriophages (Fischetti, 2010; Lood et al., 2014, 2015; Thandar et al., 2016) and predatory bacteria (Shatzkes et al., 2017a), means to specifically limit the spread of resistance through horizontal gene transfer (HGT) has not yet been addressed. In recent years, WWTPs have been identified as “hotspots” for the emergence and transmission of AMR (Manaia et al., 2018) via selective pressure (e.g., subclinical levels of antibiotics; Andersson and Hughes, 2014) and HGT, respectively. Several studies have shown that despite the majority of bacteria being removed in the water purification process, a large diversity of the antibiotic resistance genes (ARGs) can be detected both in the activated sludge and the effluent water (Calero-Cáceres et al., 2014; Bengtsson-Palme et al., 2016; Barancheshme and Munir, 2017; Zhang et al., 2018). These ARGs are found in cell-free DNA (originating from dead bacterial cells) and in bacteriophage fractions (Lood et al., 2017). Considering the impact of transformation and transduction in the spread of resistance, the fate of extracellular and phage-associated ARGs cannot be neglected. The concept of elimination of recombinant DNA from the environment by using predatory bacteria has been studied earlier by Monnappa et al. (2013). They demonstrated that *B. bacteriovorus* HD100 is able to effectively remove recombinant bacterial strains in aqueous and soil slurry environments. This, in turn, led to a reduction of the prey-associated recombinant plasmid, limiting the chances for HGT. Predatory bacteria thrive in environments with high prey density, hence are naturally occurring in WWTPs (El-Shanshoury et al., 2016; Feng et al., 2016; Yu et al., 2017). Not only do they kill the prey, but they also completely degrade its DNA (Matin and Rittenberg, 1972; Rosson and Rittenberg, 1979), consequently reducing the pool of ARGs in the environment. The repertoire of enzymes secreted by this bacterium may further contribute to the reduction of the HGT. Extracellular proteolytic and nuclease activities have been demonstrated in *B. bacteriovorus* HD100 cultures (Engelking and Seidler, 1974; Gloor et al., 1974; Monnappa et al., 2014; Bratanis et al., 2017; Bratanis and Lood, 2019). Thus, hypothetically, nucleases released into the environment may contribute to the elimination of the “cell-free ARGs,” while extracellular proteases may act on phage particles, leading to their inactivation. Such an economical and environmental-friendly application of *B. bacteriovorus* could work in tandem with *B. bacteriovorus-*stimulated sludge biolysis which has been recently suggested as a method to dewater sludge and reduce its mass (Yu et al., 2017). Based on the existing data outlined above, we and others have raised the idea of predatory bacteria as a mean to regulate such spread of AMR through the usage of BALOs (Figure 2). However, more experiments need to be conducted for such a theory to be tested.

## *B. bacteriovorus* Bioextraction of Bioplastics

In addition to the previously described potential applications, *B. bacteriovorus* has also been investigated as a lytic agent for the recovery of intracellular bio-products produced by Gram-negative bacteria. The advances in systems-biology, high-throughput omic techniques and improved computational tools are enabling enhanced *in silico* predictions of bacterial physiology, metabolic pathways and regulatory networks. This in turn allows engineering of specific metabolic pathways for industrial purposes, facilitating the development of microbial chassis for biotechnological applications such as the production of bacterial polyesters or polyhydroxyalkanoates (PHAs). PHA is a unique polyester made naturally by certain bacteria including different strains of the Gram-negative bacterium *Pseudomonas putida*. With the general increase in environmental awareness, and the problems related to waste disposal and slow degradation kinetics of the traditional petroleum-based plastics, biodegradable PHAs are becoming an increasingly interesting alternative. Some of the most widely studied producers of PHA are *P. putida* strain KT2440 and *Pseudomonas oleovorans* strain GPo1, although the biodegradable polymer is also produced in recombinant *E. coli* strains (Nikel et al., 2006; Prieto et al., 2016). As they are being produced the polymers accumulate in a form of intracellular granules in the bacterial cytoplasm, which has made the product recovery difficult and expensive. Consequently, a lot of effort has been invested in the development of efficient methods for biopolymer recovery, a key step in the production process with direct impact on profitability. One of the most established methods of product isolation is mechanical cell disruption by high-pressure homogenization (Tamer et al., 1998). Other tested methods include filtration, continuous centrifugation, enzymatic digestion or the use of detergents and solvent. Although many of these methods result in recoveries ranging from 70 to 90%, disadvantages including high costs, complicated and lengthy procedures, and environmental problems render them inapt for industrial large scale production (Yang et al., 2011; Madkour et al., 2013). In contrast to several tested phage-based methods for recovery, which are species-specific and might require some engineering to optimize, the use of *B. bacteriovorus* as a lytic agent is more generally applicable and robust, as it preys on a wide range of Gram-negative bacteria. It has been shown that *B. bacteriovorus* produces a specific extracellular depolymerase, the extracellular-like mcl-PHA depolymerase (PhaZ_Bd_), which degrades a fraction of the accumulated biopolymer. This PHA degradation results in a carbon and energy source, readily available to be utilized by bdellovibrio, conferring ecological advantages such as motility and increased predation efficiency to the predator (Martínez et al., 2013; Prieto et al., 2016). The use of *B. bacteriovorus* HD100 as a downstream tool for the recovery of intracellular bioproducts has been further optimized by bacterial engineering, by constructing a PhaZ_Bd_ knockout mutant, in order to avoid PHA degradation. Using the PhaZ_Bd_-deficient strain resulted in the recovery of >80% of the PHA accumulated in the prey cells, compared to 54–60% with the wild type strain. These results have encouraged further investigation, potentially providing a system for harvesting bioproducts such as PHA in one step, reducing the industrial use of detergents and solvents (Martínez et al., 2016).

## Genetic Tools for Modification of *B. bacteriovorus*

Future biotechnological application of *B. bacteriovorus* and other predatory bacteria may require genetic engineering of strains with desired features. Despite six decades of research on *B. bacteriovorus*, the genetic toolbox for this bacterium is still limited. Pioneering work of Cotter and Thomashow (1992a) opened the door for genetic manipulation of bdellovibrios. They identified the first cloning vectors for *B. bacteriovorus* and developed the procedure to conjugally transfer recombinant DNA from *E. coli* to *B. bacteriovorus*. The IncQ-type plasmids were found to autonomously replicate in the predator cells, while the IncP-type plasmids were maintained through integration into the genome via Campbell-like recombination. Building on this finding, Lambert et al. (2003) utilized the IncP plasmid pSET151 as a tool for creating targeted knock-outs. They successfully inserted a kanamycin cassette with flanking homology regions into *mcp2* and *mviN* genes. As no counterselection was used in this method, the frequency of a second cross-over event was relatively low and the process of screening exconjugants was laborious. Publication of a complete genome sequence for *B. bacteriovorus* HD100 (Rendulic et al., 2004) contributed to further development of reverse genetic methods. Steyert and Pineiro (2007) established a technique to create markerless deletion mutants. They constructed a suicide plasmid, pSSK10, containing a counter-selectable marker *sacB* for enrichment of excisants. *sacB* confers sucrose sensitivity, thus addition of sucrose to the growth medium effectively selects for double recombinants in *B. bacteriovorus*. Another *sacB*-based plasmid used for generation of marker-free deletions (or allelic exchange) in *B. bacteriovorus* is pK18*mobsacB* (Schäfer et al., 1994; Roschanski et al., 2011; Hobley et al., 2012a).

The complementation of mutant strains can be achieved via integration of the suicide plasmid derivatives into the chromosome (single-copy complementation) or through the use of autonomously replicating shuttle vectors (single- or multi-copy complementation). Examples of the latter include: pSUP202, pSUP404.2 (Roschanski and Strauch, 2011), pMMB206 (Flannagan et al., 2004; Steyert and Pineiro, 2007), and pPROBE-NT (Miller et al., 2000; Avidan et al., 2017).

Different set of genes are essential for HD and HI growth (Medina et al., 2008; Tudor et al., 2008; Duncan et al., 2019). Thus, HD strains can be used to inactivate genes essential for HI growth while knock-out of predation-essential genes can be achieved using HI strains. Several research groups established transposon mutagenesis protocols using facultative HI isolates of *B. bacteriovorus* (Medina et al., 2008; Tudor et al., 2008; Duncan et al., 2019). While conjugation is the most common method to deliver recombinant DNA into *B. bacteriovorus*, Tudor et al. (2008) reported that electroporation is equally efficient to introduce the transposon-containing plasmid pRL27 into HI *B. bacteriovorus* 109JA and 109J-SJ cells.

At present, a main barrier to elucidate gene functions at the molecular level is a relative deficit of tools to manipulate gene expression in *B. bacteriovorus* cells. To our knowledge, there are only two methods available thus far: (i) expression of antisense RNA, which was successfully used to downregulate predation-essential genes in the wild type obligate predator background (Flannagan et al., 2004), and (ii) synthetic riboswitches, which enabled regulated expression of the flagellar genes (Dwidar and Yokobayashi, 2017). Heterologous promoters such as P*_nptII_* and P*_lac_* from *E. coli*, were used to express *gfp* (encoding green fluorescent protein) in *B. bacteriovorus*. Constitutive *nptII* promoter was proved to be functional in both, HD and HI growth phase (Mukherjee et al., 2015). Expression of *gfp* under inducible *lac* promoter was also observed in HD and HI strains (Flannagan et al., 2004; Roschanski and Strauch, 2011), but as noted by Flannagan et al. (2004) it was independent of IPTG and could not be regulated.

## Potential Limitations

While biotechnological application of purified enzymes from predatory bacteria seems to be realistic, the application of the whole cells is likely to be more challenging. Several environmental factors such as optimal growth conditions, pollutants or microbial interactions must be considered before natural enemies can be used as a biocontrol agent in complex systems.

In a laboratory-scale experiment Feng et al. (2017) showed that exogenous addition of *B. bacteriovorus* UP (strain isolated from activated sludge at Ulu Pandan Water Reclamation Plant, Singapore) to the activated sludge significantly altered the composition of the microbial community. These perturbations result from indiscriminative predation and can be detrimental to the activated sludge process. However, as noted in the same paper, results of the small-scale experiment may not reflect the situation in a full-scale reactor. It is unknown how, e.g., the temperature or periodic anoxic conditions in wastewater treatment process affect the behavior of bdellovibrios. Also, it is possible that any changes in microbial community structure would be able to recover over a longer time period. The level of oxygen is proven to be critical for *B. bacteriovorus* ability to prey upon cells. While maintaining the ability to reduce biofilms, lack of oxygen inhibits any predation upon planktonic cells (Kadouri and Tran, 2013; Patini et al., 2019).

Bdellovibrios are very sensitive to various environmental pollutants, which in turn affect their predatory activity (Wehr and Klein, 1971; Varon and Shilo, 1981; Markelova, 2002). Studies on susceptibility to phenol and urea showed that both of these common wastewater toxicants affect *B. bacteriovorus* life cycle (Markelova, 2002). The presence of urea or phenol reduced the number of viable cells in the liquid culture, but the toxic effect was lower when *B. bacteriovorus* was attached to the surface (i.e., associated with biofilm). Markelova also suggested that under unfavorable conditions *B. bacteriovorus* cells are able to persist inside surface-attached bdelloplasts, which protect them from the environment. The formation of stable bdelloplasts as a survival strategy was in agreement with previous studies by Sánchez-Amat and Torrella (1990). Another wastewater pollutants that might have an impact on Delta-BALOs are surfactants. It has been demonstrated that *B. bacteriovorus* and *Peredibacter starrii* are very sensitive to sodium dodecyl sulfate (SDS) (Cho et al., 2019), which is widely used in detergents and personal care products. The viability of both, free-swimming AP cells and those within bdelloplasts, is drastically affected by the low concentrations of this organic compound; whereas the prey population is not affected. Such selective effect of SDS might be applied to selectively terminate undesirable predation without affecting the viability of the prey.

Agricultural application of BALOs can be affected by the presence of herbicides that are widely used for weed control. Wehr and Klein evaluated the effect of 17 different herbicides for activity against *B. bacteriovorus* (Wehr and Klein, 1971). The plaque formation was inhibited, to various degree, by 11 herbicides included in the analysis. The phenylurea herbicide linuron showed the strongest inhibitory effect, and it was proven to have lethal effect on *B. bacteriovorus* cells.

Despite of preying on wide range of host bacteria, BALOs might have different effects on various species in mixed microbial communities. As stated by Rogosky et al. (2006), *B. bacteriovorus* displays preferential predation of the favored prey. The basis for this selection is not known, but might be a result of rapid attachment to the preferred species. Thus, using BALOs as biocontrol agent in complex environment may risk that the target prey would not be the preferred one. Finally, possible emergence of HI mutants of *B. bacteriovorus* might be problematic for biotechnological applications. They not only exhibit reduced predation ability but may also contribute to formation of undesired biofilm.

## Conclusion

**FIGURE 3:**
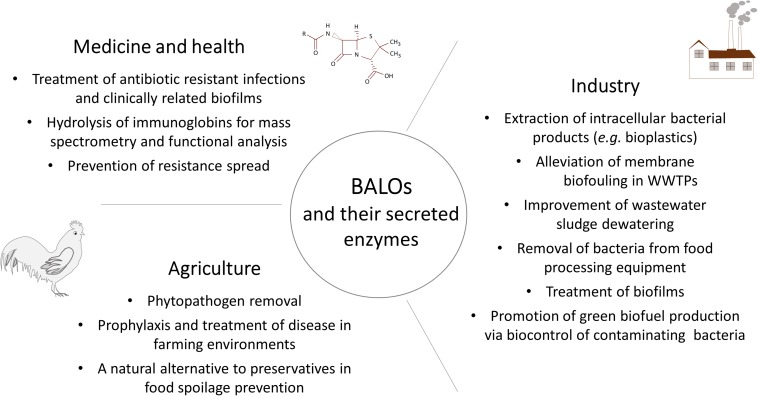
Overview of BALOs biotechnological applications discussed throughout the review.

Due to their natural ability to eliminate Gram-negative bacteria, BALOs have great potential as a biocontrol agents for both planktonic and biofilm bacteria (Figure 3). At present, the main obstacle to put this idea into practice is the lack of sufficient knowledge about the ecology of predatory bacteria. Most of the published studies use pure cultures to elucidate prey-predator interaction. However, it has been demonstrated that the presence of even single decoy influences predation efficiency (Hobley et al., 2006). What is the predatory behavior of BALOs in complex natural habitats, is still to be discovered. The plethora of hydrolases produced by predatory bacteria may serve as a source for exploring new biotechnologically relevant enzymes (Figure 3); an area that needs further research to evaluate its full potential. Although a number of fundamental properties underlying *B. bacteriovorus* predation have been revealed, and our understanding of this bacterium and its intriguing lifestyle is improving, it is evident that much work remains to be done before we have achieved a comprehensive understanding of this ubiquitous, and clearly very versatile predator. Nevertheless, that’s not an obstacle for applicative R&D.

## Author Contributions

All authors contributed to the drafting and writing of the manuscript, and critically reviewing the final manuscript. All authors approved the final version of the manuscript.

## Conflict of Interest

RL is employed by Genovis AB, a company developing biotechnological tools for analysis of immunoglobulins. The remaining authors declare that the research was conducted in the absence of any commercial or financial relationships that could be construed as a potential conflict of interest.
